# Varicella susceptibility and transmission dynamics in Slovenia

**DOI:** 10.1186/1471-2458-10-360

**Published:** 2010-06-23

**Authors:** Maja Sočan, Nataša Berginc, Jaro Lajovic

**Affiliations:** 1 Centre for Communicable Diseases, National Institute of Public Health of Republic of Slovenia, Trubarjeva 2, 1000 Ljubljana, Slovenia; 2 Laboratory for virology, Centre for Communicable Diseases, National Institute of Public Health of Republic of Slovenia, Bohoričeva 15, 1000 Ljubljana, Slovenia; 3 Rho Sigma Research & Statistics, Topniška 45, 1000 Ljubljana, Slovenia

## Abstract

**Background:**

A cross-sectional, age-stratified study was conducted to determine varicella-zoster seroprevalence and force of infection in Slovenia.

**Methods:**

3689 serum samples were tested for VZV IgG antibodies with an enzyme immunoassay. Semiparametric and parametric modelling were used to estimate the force of infection.

**Results:**

Overall, 85.6% of serum samples were seropositive. Age-specific prevalence rose rapidly in preschool children and over 90% of 8 years old tested positive for VZV. However, 2.8% of serum samples among women of childbearing age were seronegative. Semiparametric modelling yielded force of infection estimates of 0.182 (95% CI 0.158-0.206), 0.367 (95% CI 0.285-0.448) and 0.008 (95% CI 0.0-0.032) for age groups 0.5- < 6, 6-11 and ≥12 years, respectively, and 0.175 (95% CI 0.147-0.202), 0.391 (95% CI 0.303-0.480) and 0.025 (95% CI 0.003-0.046) for age groups 0.5- < 5, 5-9 and ≥10 years, respectively.

**Conclusions:**

Regardless of the age grouping used, the highest transmission occurred in children in their first years of school.

## Background

Varicella is among the most common vaccine-preventable childhood diseases in countries with a temperate climate. In preschool and school-aged children it is generally considered to be a mild, self-limiting disease [1]. In newborns, immunocompromised patients, adults and pregnant women, however, it can lead to severe complications such as acute cerebellar ataxia, encephalitis and varicella pneumonia [2-4]. Varicella pneumonia is the most common complication in adults, requiring hospitalisation in approximately one in 4000 varicella cases [1]. Infection of pregnant women, especially in the first three months of gestation, can cause congenital varicella syndrome in the newborn [5,6].

With a clinical attack rate of 65-85% following a household exposure of susceptible individuals, varicella is a highly contagious disease. Varicella-zoster virus, the causative agent of varicella (and herpes zoster), is prevalent worldwide, but its epidemiological features differ in temperate versus tropical climates. In temperate regions, varicella is largely a childhood disease with a rapid acquisition of antibodies in the early years of life [7-16], while in the tropics, it mostly occurs in adolescence or adulthood [17,18].

Also, the VZV-specific antibodies seem to appear much earlier in some European countries than in the others - differences which are difficult to explain [16]. Dissimilarities in environmental determinants (e.g. climatic factors) and distinctive social structures may affect the seroepidemiology of VZV.

The varicella vaccine was developed over thirty years ago. In 1998, the WHO recommended vaccination against varicella as a part of routine vaccination schedules in countries where varicella represents an important public health problem and socio-economic burden [19]. The WHO recommendations have been adopted by some economically developed countries, including USA and Australia that could afford the implementation of a varicella vaccination programme. Most European countries recommend varicella vaccination only for high-risk groups. Universal routine varicella vaccination programmes were instituted in Germany, Greece, certain regions of Spain and Italy, but not elsewhere [20,21]. The reason for not implementing routine varicella vaccination programmes might be the lack of recognition of varicella as a serious disease. A concern has been raised that widespread childhood varicella vaccination will shift the peak incidence of the disease from children to young adults and result in higher numbers of severe cases [20]. Mathematical modelling suggested that routine varicella vaccination might generate an upward trend in herpes zoster incidence at least in the short to medium term [22,23].

Implementation of the universal varicella vaccination programme in the USA was followed by a rapid decline in incidence of varicella among immunized children, associated with a cohort effect in the non-immunized population. The initially recommended one-dose schedule provided only limited protection as a result of primary and/or secondary failures, i.e., waning vaccine-derived immunity. In 2007, the ACIP (Advisory Committee on Immunization Practices) advised that the one-dose schedule be replaced by a two-dose schedule in the USA. The interval between the first and the second dose may be short (but should be at least one month), standard (3 to 7 years apart), or even longer, depending on varicella epidemiology [24]. Thus, the decision concerning an optimal vaccination schedule depends greatly on the local epidemiological situation.

Slovenia is among the countries where a routine varicella vaccination programme has not yet been implemented. The present study was designed to investigate the Slovenian epidemiological situation in order to aid planning of the optimal varicella vaccination strategy in Slovenia. For this purpose, a cross-sectional survey of the seroprevalence of VZV antibodies was performed and the collected data were used to assess the force of VZV infection.

The force of infection is a central parameter in the assessment of an infection within a population. The force of infection is the rate at which susceptible individuals become infected by an infectious disease, whilst taking susceptibility into account. The measure can be used to compare the rate of transmission between different groups of the population for the same infectious disease. It depends on a variety of age-dependent factors, is thus itself age-dependent and, therefore, provides the key information regarding childhood vaccination schedule.

## Methods

### Seroprevalence survey

For the cross-sectional, age-stratified seroprevalence survey, a total of 3,689 sera from individuals aged 0-97 years were tested for VZV IgG antibodies. Serum samples were collected at the Clinical Institute of Clinical Chemistry and Biochemistry Ljubljana in 2006 using residues from specimens taken for routine diagnostic tests.

The overall age structure of the total sample was chosen according to the ESEN (European Seroepidemiological Network) specifications: about 100 samples were obtained per each of the 20 age groups 0-19 years and about 200 samples per each of the 7 age groups ≥20 years (20-24, 25-29, 30-34, 35-39, 40-49, 50-59, ≥60) with approximately equal number of samples by gender in each of the groups [25].

The anonymised samples, marked only with the date of sampling, age and sex of the patient, were sent to the Laboratory for Virology at the Institute of Public Health where varicella antibody assays were performed.

The sera were stored at -20°C before being tested with a commercial enzyme-linked immunoassay (ELISA; Enzygnost Anti-VZV Virus/IgG, Dade Behring, Marburg, Germany) that was used for the determination of specific IgG antibodies against VZV. The assay was run in accordance with manufacturer's instructions. Optical density measurements were performed and results were calculated using an automated system (Dade Behring BEP III System, Dade Behring, Marburg, Germany). All tests were evaluated using adequate positive and negative reference controls according to the manufacturer's instructions. Results were classified as positive, negative or equivocal. Specimens with equivocal results were retested using the same test kit and those few that were equivocal again were classified as negative [26].

The protocol for the seroprevalence survey was approved by the National Medical Ethics Committee of the Republic of Slovenia.

### 2.2. Estimation of the force of infection

Two different approaches were employed to estimate the force of VZV infection based on our data. The first approach was based on a semiparametric model (piecewise constant force of infection) as described by Mossong [27]; this approach also provided the possibility of direct comparison with some recent results reported for other European countries [16]. The second approach was based on the use of a parametric (catalytic) model as reported by Farrington and based on the work of Griffiths [28,29]. Such a two-tier combined evaluation seemed appropriate, as both semiparametric and parametric methods have limitations [30].

A detailed description of the modelling is given in Additional file 1.

All modelling and data analyses were performed using the R statistical software package version 2.9 [31].

When estimating the force of infection based on serological data, some basic assumptions are made and certain methodological issues have to be considered. These are covered elsewhere, but two issues should be noted here [29,32,33]. Firstly, inherited maternal antibodies, which yield a high proportion of seropositive infants, lead to low force of infection in the early months of life. Exploratory analyses with our data showed the age group <0.4 yr (~5 months) to be associated with practically 100% seropositivity. This is in accordance with the general notion of the average maternal antibody protection of about 6 months. To avoid this confounding factor, data from infants <6 months of age (N = 73) were excluded from further analysis. The second issue is brought about by the fact that our data do not represent a random sample from the population. This is not an uncommon feature of seroepidemiological studies, but the issue should be taken into consideration when interpreting the results [16].

## Results

### Serological testing

Overall, 3,158 of 3,689 (85.6%) sera tested positive for varicella IgG antibody; the percentage of positive results was similar between males and females (83.2% vs. 87.5%, respectively).

The positivity rate for varicella antibodies was high in the first months of life - IgG antibodies were detected in 81% of children less than 6 months of age. This passively acquired immunity, however, did not persist long and the percentage of IgG-positive children was only 16% in the age group 6-12 months. Presumably some children in this age group have already acquired natural varicella immunity.

By their fifth year of age, more then half (56.2%) of the children tested seropositive for VZV. Seropositivity increased with increasing age and, at 8 years of age, VZV antibodies were detected in 90% of children. The rise in seropositivity was moderate in teenagers. The trend was similar for males and females.

There was still a 4% seronegative group of individuals in young adulthood. Also, there was a relatively large proportion (2.8%) of seronegative subjects among women of childbearing age (15-49 years).

### Semiparametric estimation of force of VZV infection

Table 1 shows the estimates of the force of infection (λ) for the Slovenian sample using two different age groupings: (a) 0.5- < 6, 6-11 and ≥12 years, and (b) 0.5- < 5, 5-9 and ≥10 years. In both cases the limits were selected to allow a direct comparison of our results with the results of two recent reports [16,27].

**Table 1:** Estimates of force of infection λ (and 95% CI) in the Slovenian sample with two age groupings: (a) 0.5- < 6, 6-11 and ≥12 years; (b) 0.5- < 5, 5-9 and ≥10 years

Age grouping (group limits)	λ	95% CI
a (6, 12 yrs)	λ_1 _0.182	0.158-0.206
	λ_2 _0.367	0.285-0.448
	λ_3 _0.008	0-0.032
b (5, 10 yrs)	λ_1 _0.175	0.147-0.202
	λ_2 _0.391	0.303-0.480
	λ_3 _0.025	0.003-0.046

Among the limitations of the model, assuming a piecewise constant force of infection for the different age groups, is that it may yield unrealistic results for certain age groups. For example, with our data it yielded a value of λ = 0 in the age group 12-18 years (grouping used: <3, 3-6, 6-12, 12-18, 18-26, >26 years), thus reproducing the problem reported by Ogunjimi [34]. Therefore, a parametric non-linear method was employed as an additional means of estimation.

### Parametric estimation of force of VZV infection

**Figure 1:**
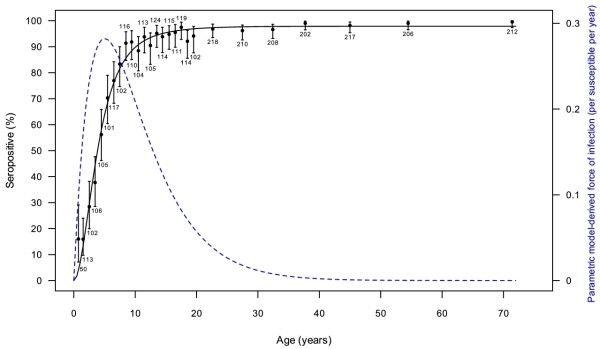
**Observed vs predicted VZV seroprevalence rates for Slovenian data (predictions generated by the parametric model; see text)**. Observed rates are shown in black dots along with 95% binomial confidence intervals. The numbers below/above the CI lines represent the total number of subjects in each age group. Proportions of seropositives predicted by the model are shown in solid line. The dashed line is the model-derived force of infection curve (the corresponding scale is on the right; λ_max _0.282 at 5.0 years).

The parameters of the model were estimated by the non-linear least squares method using the R statistical package, and the goodness-of-fit was evaluated by calculating the deviance. The overall fitting of the parametric model is good (deviance 0.031, residual standard error 0.035 on 25 d.f.), which is also supported by Figure 1. This allows for the model-based estimation of the force of infection, as the hazard of infection can be calculated as a negative logarithm of the derivative of the survival function yielding the following formula:

The formula comes from a procedure of model selection (equation 6, Additional file 1). From the practical standpoint, its parameters can be interpreted as determining the maximum force of infection (parameter a) and the age at the maximum force of infection (reciprocal of parameter b). A graphical representation of the data and the fitted force of infection curve are shown in Figure 1 and the parameter estimates are presented in Table 2. Using this model, the calculated point estimate of the peak force of infection is 0.282 (95% CI: 0.228-0.353) at 5.0 years of age (95% CI: 4.3-6.0). Estimates of λ by age groups may be obtained by integrating the above function over the appropriate interval, divided by the interval length.

**Table 2:** Parameter estimates of the fitted parametric model [equation 6, Additional file 1]

Parameter	Value	SE
a	0.153	0.012
b	0.199	0.016

## Discussion

According to the Slovenian National Immunisation Programme guidelines, varicella vaccine is only recommended for individuals at the highest risk of complications, e.g. for immunocompromised subjects if their immune status permits the application of live, attenuated varicella vaccine. Also, the varicella vaccine is recommended for all those who reach adulthood without having been infected with varicella-zoster virus, particularly for people with a professional risk for such infection (health care workers, kindergarten staff etc.). In practice, these broad recommendations are not followed and only a small proportion of people actually receive the vaccine. The annual vaccination report data (electronic dataset, National Institute of Public Health) show that less than 100 people are vaccinated against varicella each year.

Due to the low number of vaccines, these vaccinations have no impact on the national varicella epidemiology or seroepidemiology. Introduction of the varicella vaccine to the national childhood immunisation programme has already been under consideration for some time. The serosurvey presented in this paper has been conducted to elucidate the seroepidemiology in Slovenia in the prevaccination period. As the tested sera were residues of specimens taken for routine diagnostic tests, they do not represent a random sample, which is an inherent weakness of many seroprevalence studies. However, varicella is so prevalent a disease that the estimates of seropositivity based on either systematically collected or residual samples yield similar results [35].

In our study, 80% of infants aged 4 months or less were VZV seropositive. Our results reconfirm the known fact about newborn infants receiving maternal VZV antibodies. This passive neonatal immunity is of a short duration as the half-life of passively acquired maternal immunoglobulins is only 6 weeks [36]. The gradual loss of maternal antibodies has already been documented for other infectious diseases, including measles, mumps and rubella. The rapid loss of protection renders children highly susceptible to various infectious diseases including varicella [1,36]. In Slovenia, the duration of maternity leave is 12 months (one month before the expected date of birth and 11 months afterwards) and children enter kindergarten from one year of age onward. Therefore, infants' siblings are probably the most common source of infections in the first year of life. In our study, only 16% of infants from 5 to 12 months of age had detectable antiVZV antibodies; most probably these were acquired by natural infection. Other studies have reported similar proportions of seropositive children in this age group [1,36].

In Slovenia, the percentage of children attending kindergarten is constantly rising. In the past, the increase in the number of children attending kindergarten was observed mainly among children from three years on and up to entering elementary school [37]. In the last few years, however, the number of children attending kindergarten has also been increasing in the age group of 1-3 years. According to national statistics, slightly more than two thirds of preschool children attended kindergarten in the school year 2008/09. The percentage of preschool children aged less than 3 years and 3-6 years who attended kindergarten was 44% and 82%, respectively. The high proportion of children having out-of-home social contacts in the first years of life may partially explain the rapid rise in VZV seropositivity. Approximately 70% of children had VZV antibodies before they entered their first year of primary education. The proportion of seropositive preschool children in our study is similar to those observed in other countries with a temperate climate. According to the ESEN2 study, over 50% of young children had antibodies to VZV by 5 years of age, with few exceptions - there were less then 40% of seropositive children in Italy and more then 80% in the Netherlands and Belgium [16]. In some countries, the infants are cared for in nurseries and other child-care facilities [16]. The early commencement of group childcare probably influences the seropositivity rate in a geographic region.

European countries collect data on vaccine-preventable diseases through paediatric sentinel systems or, less frequently, through obligatory reporting systems. Slovenia is among those rare European countries where varicella has been a mandatory notifiable disease for over 20 years. As many as 75% of varicella cases reported were pre-school children, the age group of 3 to 4 years being the most affected. Age-specific distribution of varicella cases showed a downward trend for all age groups except for children younger than 4 years. In this age group, the incidence rate demonstrated an evident upward trend [38]. However, these changes could also theoretically derive from modification in reporting - e.g. a general increase in under-reporting over time. The obligatory notification system has been assessed for completeness and consistency recently [39]. The average rate of notified cases was 76.2% in last ten years when compared to health statistics data. The mandatory notification system in Slovenia provides enough information to survey age/sex-specific varicella trends in the prevaccine era [39]. Kindergarten attendance is probably one of the social determinants for the high number of varicella cases and high seroprevalence in preschool children in Slovenia. The number of siblings also determines varicella susceptibility. One study showed that individuals growing up without a sibling or with one sibling were most likely to be seronegative [40]. In Slovenia, the low number of children per family (37% of families having only one child) could have a negative effect on varicella seropositivity.

There is a lack of varicella immunity in teenagers, and a small proportion of young adults, aged from 19 to 34 years, are still seronegative. The decreasing proportion of seronegatives (from 5.9% to 3.2%) in this age group shows that infection (most probably associated with a clinically apparent disease) still occurs, although the likelihood of coming in contact with VZV is decreasing.

In our study, the highest force of infection (FOI) has been observed in the 6 to 11 or the 5 to 9 years groups (0.361 and 0.391, respectively), estimated by the semiparametric method. The parametric method identified the highest force of infection (0.282) at age 5 (Figure 1). The VZV FOI in Slovenia is comparable to Germany, Finland and Spain for all three age groups (<5, 5-9, >10 years) [16]. Compared to our results, the FOI for children <5 was much higher in the Netherlands, Belgium and Luxembourg. The preschool mixing pattern is the major determinant for a high FOI. A study compared two groups of children - a group that entered all-day nurseries or kindergartens early and a group that entered late [41]. The FOI in the first group was much higher than in the latter. The social behavior and climatic determinants deserve detailed study in the future to improve our understanding of varicella epidemiology in the prevaccination and postvaccination periods.

## Conclusion

In conclusion, our data show that the most appropriate approach to reduce the burden of varicella would be a universal, standard two dose varicella vaccination programme (the first dose administered at 12-18 months and the second at 3-6 years) [20]. After the introduction of varicella vaccine in the childhood immunisation schedule, a vigilant surveillance of varicella epidemiology and seroepidemiology should continue in order to promptly identify gaps and unexpected epidemiological changes in both varicella and herpes zoster.

## Competing interests

The authors declare that they have no competing interests.

## Authors' contributions

MS conceived and designed the study, organised serological data collection, managed the overall project coordination and drafted the major portion of the manuscript. NB participated in the serological analysis and interpretation and drafted the respective section of the manuscript. JL performed mathematical modelling, programming and statistical analysis and drafted the respective sections of the manuscript. All authors read and approved the final manuscript.

## Pre-publication history

The pre-publication history for this paper can be accessed here:

http://www.biomedcentral.com/1471-2458/10/360/prepub

## Supplementary Material

Additional file 1 **Modelling of force of VZV infection**. Additional file 1 contains a detailed description of semiparametric and parametric estimation of force of VZV infection. Click here for file
