# Supplementary material for: Varicella susceptibility and transmission dynamics in Slovenia
Source: BMC Public Health. 2010 Jun 23;10:360. doi: 10.1186/1471-2458-10-360 (PMC2901375; doi:10.1186/1471-2458-10-360)
Supplement: Additional file 1 — Modelling of force of VZV infection. Additional file 1 contains a detailed description of semiparametric and parametric estimation of force of VZV infection. [file 1471-2458-10-360-S1.DOC]

**Additional file 1**

Although data in this type of serological study are obtained by cross‑sectional sampling, they are routinely treated as if being longitudinal. From the epidemiological standpoint, the following developments are consistent with a simple age-structured SIR transmission model at endemic equilibrium [1]. From the analytical standpoint, the (decreasing) empirical distribution of seronegativity is interpreted as a survival function, with the number of seropositive subjects observed in each age group/age band being treated as a binomial variable with the probability density function:

P (*X* = *k*) = $\left(\begin{matrix} n \\ k \end{matrix}\right)$ F(*x*)*^k^* · (1–F(*x*))*^n–k^* (1)

where *n* is the total number of subjects sampled in an individual age group, *k* is the number of seropositive subjects in that age group (with the difference *n*–*k* thus corresponding to the number of seronegative individuals), and F(*x*) is the average proportion of seropositive subjects at the mean age *x_i_* in the respective age group (it should be noted that F(x) is not an observed quantity but a predicted one.) The proportion may be expressed as:

F(*x_i_*) = 1 – exp (–$\sum_{j=1}^{i} \lambda_{j}$) (2)

where λ_j_ is the force of infection in the age group (with the corresponding survival function, i.e., the proportion of seronegative subjects, being S(*x*) = 1 – F(*x*)). These basic relationships allow the estimation of joint age-specific forces of infection, detailed below. The force of infection was assumed to be 0 for ages below the maternal antibody protection threshold, i.e., 0.5 yr in our case. The first step of the present analysis was the estimation of age-dependence of force of VZV infection and assessment of the age corresponding to its maximum.

# *(a) Semiparametric estimation of force of VZV infection*

The estimation was made assuming a piecewise constant force of infection [2, 3] and based on the model as described by Mossong and obtained under the same assumptions [4]. It is derived from equations (1) and (2), defining the F(*x*) – the proportion of seropositive subjects – in the age group *i* as

F(*x_i_*) = $1-\exp\left(-\lambda_{i}(x_{i}-m_{i})+\sum_{j=1}^{i-1} \left[-\lambda_{j}(m_{j+1}-m_{j})\right]\right)$ (3)

where *i* is the age group, λ_i_ the force of infection, *x_i_* the mean age in the age group *i*, and *m_i_* the lower limit of the age group *i* (with *m_1_* being formally set to 0.5, i.e., the average duration of protection by maternal antibodies).

The values of the parameters *λ_i_* are obtained by the maximum likelihood estimation using maximization of the kernel of the likelihood function, L:

L = $\sum_{j=1}^{g} k_{j}\log(F(x_{j}))+(n_{j}-k_{j})\log(1-F(x_{j}))$ (4)

where *g* is the number of age groups, *k_j_* is the number of seropositive subjects, *n_j_* is the total number of subjects in the age group *j*, and F(*x_j_*) is as in eq. (3).

*(b) Parametric estimation of force of VZV infection*

Age-specific force of infection can be effectively estimated by modelling F(*x*) based on the so-called catalytic model. F(*x*) of childhood diseases has been shown to be well fitted by the equation:

F(x) = 1 – $\exp\left(\frac{a}{b}xe^{-bx}+\frac{1}{b}\left[\frac{a}{b}-c\right]\left[e^{-bx}-1\right]-cx\right)$ (5)

where the meaning of *x* and F(*x*) is as explained for equation (1), while *a*, *b* and *c* are the parameters to be estimated (*a*, *b*, *c* ≥ 0).

After a graphical validation of the appropriateness of the model for our data, the model was fitted using the nonlinear least squares method. The parameters *a* and *b* were both highly significant, while *c* did not differ significantly from zero (implying that the force of infection is asymptotically declining to zero). As the model including all 3 parameters (*a*, *b* and *c*) did not differ significantly from the model with only 2 parameters (*a* and *b*) (F = 0.09, p = 0.76), the parameter *c* was omitted, yielding the final model:

F(x) = 1 – $\exp\left(\frac{a}{b}xe^{-bx}+\frac{a}{b^{2}}\left[e^{-bx}-1\right]\right)$ (6)

Anderson RM, May RM: **Infectious Diseases of Humans: Dynamics and Control.** Oxford, New York: Oxford University Press, 1992.

Becker NG: **Analysis of Infectious Disease Data.** London, New York: Chapman and Hall, 1989, 196.

Gail MH, Benichou J: **Encyclopedia of Epidemiologic Methods.** Chichester: John Wiley & Sons; 2000, 220-221.

Mossong J, Putz L, Schneider F: **Seroprevalence and force of infection of varicella-zoster virus in Luxembourg.** *Epidemiol Infect* 2004, **132**:1121-1127.
